# Rapid electrochemical sensor for uranium(vi) assessment in aqueous media

**DOI:** 10.1039/d2ra02619h

**Published:** 2022-07-11

**Authors:** Zeinab F. Akl

**Affiliations:** Egyptian Atomic Energy Authority P.O. Box 11762 Cairo Egypt eltasneem2007@yahoo.com

## Abstract

The significance of reliable monitoring of uranium levels in water recourses calls for the development of time-saving, robust, and accurate methods for its estimation. In this view, the current study describes the design and analytical parameters of a potentiometric membrane sensor for uranium(vi) ions. The sensor is based on a new Schiff base derivative, as an ionophore, that was synthesized and structurally characterized by elemental, FTIR, and ^1^HNMR analyses. The impact of the membrane constituents was studied and the membrane composition of PVC (32.50) : *o*-NPOE (65.00) : ionophore (2.00) :  KTpClPB (0.50) (%, w/w) achieved the optimal performance. A Nernestian response was observed for uranium(vi) ions within the concentration range 1.00 × 10^−6^ to 1.00 × 10^−1^ mol L^−1^. The sensor revealed a low detection limit of 3.90 × 10^−7^ mol L^−1^ with satisfactory reproducibility. Stable and reproducible potentials were obtained within a short time (9 s) over the pH range 2.10–4.21. The impact of possible competing ions was investigated and the selectivity coefficients revealed appropriate selectivity for uranium(vi) ions over various cations without significant interference. The sensor's performance was examined by determining the amount of uranium(vi) in water samples and the results showed no significant differences from those obtained by the ICP-OES method.

## Abstract 2

A new Schiff base was synthesized and applied as ionophore to construct potentiometric sensor for uranium(vi) determination.

## Graphical abstract

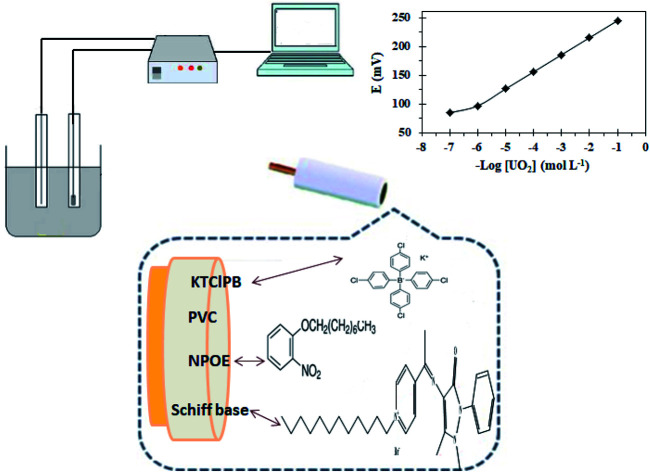

## 1. Introduction

The cumulative effects of excessive uranium levels in the environment endanger human beings and aquatic life,^1^ accordingly, uranium monitoring and assessment in real samples has become a growing concern. Natural and anthropogenic uranium contamination can induce severe toxicological and radiological risks;^2^ as a result, rapid and sensitive detection of uranium and uranium-bearing compounds is highly demanded for many civilian and military applications. Compared to conventional uranium detection techniques, electrochemical sensors have some attractive attributes such as user-friendly operation, short examination time, applicability to low sample volume, economic efficiency, durability, good sensitivity, and on-site applications.^3^ As such, the electrochemical recognition of uranium has recently emerged as a significant analysis method for the nuclear industry and environmental monitoring.^4^ Accordingly, an increased number of various electrochemical sensors were developed for uranium(vi) ions to meet the growing analysis demands of diverse samples.^5^

Potentiometric sensors are a well-established, rapidly growing subgroup of electrochemical sensors which are characterized by flexibility, good precision, wide operating range, low maintenance, and low-energy consumption.^6,7^ Additionally, the potentiometric analysis doesn't alter the chemical composition of the measured sample which allows for its further analysis by other techniques, if required.^8^ In recent years, potentiometric sensors have become a successful tool for selective and sensitive routine analysis of a wide variety of chemical species for environmental, industrial, and clinical applications.^9^ The key piece of a potentiometric sensor is the sensing component or ionophore that should be selected carefully to enhance the sensor interaction towards the target species and consequently the selectivity behavior.^10^ From this perspective, the synthesis of new ionophores is of continued attention. The ionophore is usually a ligand with the ability to preferentially bind analyte ions where its nature affects the stability and stoichiometry of the formed ionophore-ion complex.^11^ Usually, compounds with multiple donor sites, for instance, N, S, and O are preferred as ionophores for chemical sensors.^12^

The utility of potentiometric sensors in uranium quantification has undergone a noticeable revolution over the last decades in view of the deadly threat that uranium poses to human health. Extensive efforts have been made to develop potentiometric uranium sensors using different compounds, such as calixarenes,^13,14^ crown ethers,^15^ organic phosphorus compounds,^16^ dimethylsulphoxide,^17^ and 2-thenoyltrifluoroacetone^18^ as ionophores. The literature reveals also the successful application of various nitrogen-based ionophores in constructing uranium selective electrodes including macrocyclic diamides,^19^ triethylenetetramine,^20^ and Schiff bases.^21–24^ Nevertheless, the design of selective and sensitive sensors to detect uranium ions is still desired.

Schiff bases have attracted much interest as convenient ionophores for various potentiometric sensors due to their high coordination capability, availability, variability in structural design, and ease of synthesis.^25^ Electrochemical sensors based on Schiff bases have revealed noteworthy sensitivity, reproducibility, selectivity, and stability for the quantification of various metal ions.^26,27^ Schiff bases, having the RCH = NR formula, are the nitrogen counterparts of aldehydes or ketones, however, the C=O group is substituted with an imine or azomethine moieties with high coordinative ability to metal ions through the nitrogen atom.^27^ The Schiff bases' structure provides an appropriate geometrical configuration and cavity size for host–guest binding of target metals resulting in good sensitivity and selectivity. Additionally, Schiff bases can stabilize many cations with various oxidation numbers thus governing their behavior in many applications including electrochemistry, antimicrobial activity, and environmental chemistry.

Schiff bases have attracted considerable attention as potential ionophores to detect uranium(vi) ions due to their selective affinity, quick exchange kinetics, and good lipophilicity. For example, 2,2′-[1,2-ethandiyl bis(nitriloethylidene)]bis(1-naphthalene),^21^*N*,*N*′-(propylenedioxy)benzenebis(salicylideneimine),^22^*N*,*N*′-4,5-(propylenedioxy)benzenebis(3,5-di-*tert*-butylsalicylideneimine),^22^*N*,*N*′-4,5-(ethylenedioxy)benzenebis(salicylideneimine),^23^ bis(2-hydroxyacetophenone)ethylenediimine,^24^ and *N*,*N*′-bis(salicylidene)-2-hydroxyphenylmethanediamine^28^ have been used as chelating agents for the electrochemical determination of uranium. The reported advantages of Schiff bases have motivated the author to design a new uranium(vi) sensor using this type of metal receptors. To continue the efforts in this direction, the successful application of a synthesized Schiff base derivative as an ion-recognition element to construct potentiometric uranium(vi) sensor is reported. The electrochemical performance and analytical applications of the developed sensor were investigated. The results showed that the developed sensor is a promising tool for uranium(vi) measurements in water samples.

## 2. Experimental

### 2.1. Chemicals and materials

The chemicals used in this study were of analytical grade. 1-(Pyridin-4-yl)ethan-1-one, 1-bromododecane, 4-amino-1,5-dimethyl-2-phenyl-1,2-dihydro-3*H*-pyrazol-3-one, *o*-nitrophenyl octyl ether (*o*-NPOE), dioctyl sebacate (DOS), chloronaphthalene (CN), dioctyl adipate (DOA), dioctyl phthalate (DOP), potassium tetrakis(4-chlorophenyl) borate (KTpClPB), and dibutyl phthalate (DBP) were obtained from Sigma-Aldrich. Tetrahydrofuran (THF), ethanol, and polyvinyl chloride (PVC) were purchased from Merck. Deionized water was applied throughout to prepare all aqueous solutions. Nitrate salts of cations were used for selectivity studies.

### 2.2. Synthesis and characterization of the ionophore

**Scheme 1:**
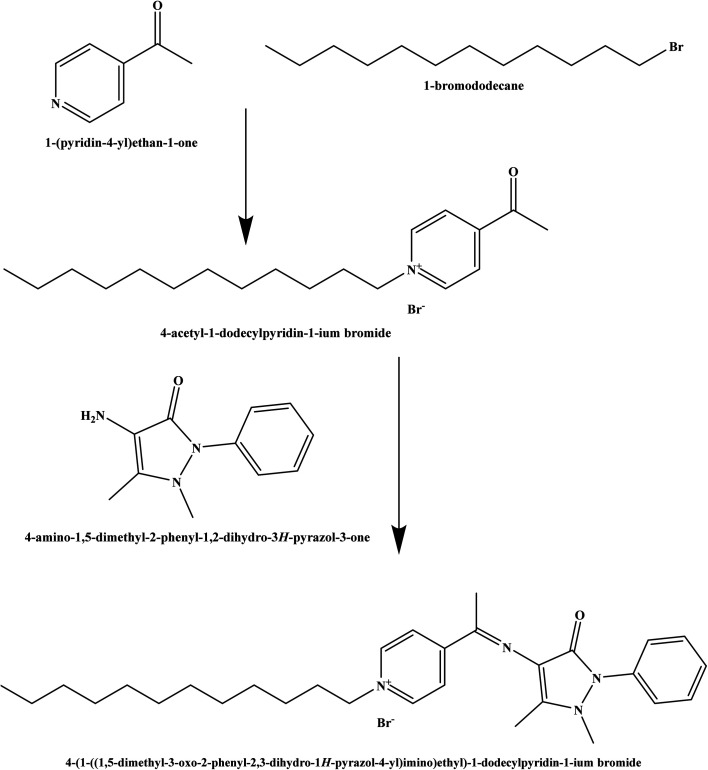
The structure of the synthesized Schiff base.

The Schiff base ionophore was prepared through two steps reactions. First, quaternization of 1-(pyridin-4-yl)ethan-1-one and 1-bromododecane in the presence of absolute ethanol as a solvent. The product was washed by diethyl ether three times, recrystallized by absolute ethanol, and then subject to a condensation reaction with 4-amino-1,5-dimethyl-2-phenyl-1,2-dihydro-3*H*-pyrazol-3-one at 80 °C for 4 h. The resulted ionophore, 4-(1-((1,5-dimethyl-3-*oxo*-2-phenyl-2,3-dihydro-1*H*-pyrazol-4-yl)imino)ethyl)-1-dodecylpyridin-1-ium bromide (Scheme 1) was dried by under vacuum to remove the ethanol and water (yield 93.7%). Elemental analysis, % for (C_30_H_43_N_4_OBr); calculated C: 64.85, H: 7.80, N: 10.08, O: 2.88, Br: 14.38; found C: 64.71, H: 7.90, N: 10.02, O: 2.97, Br: 14.40.

**Fig. 1:**
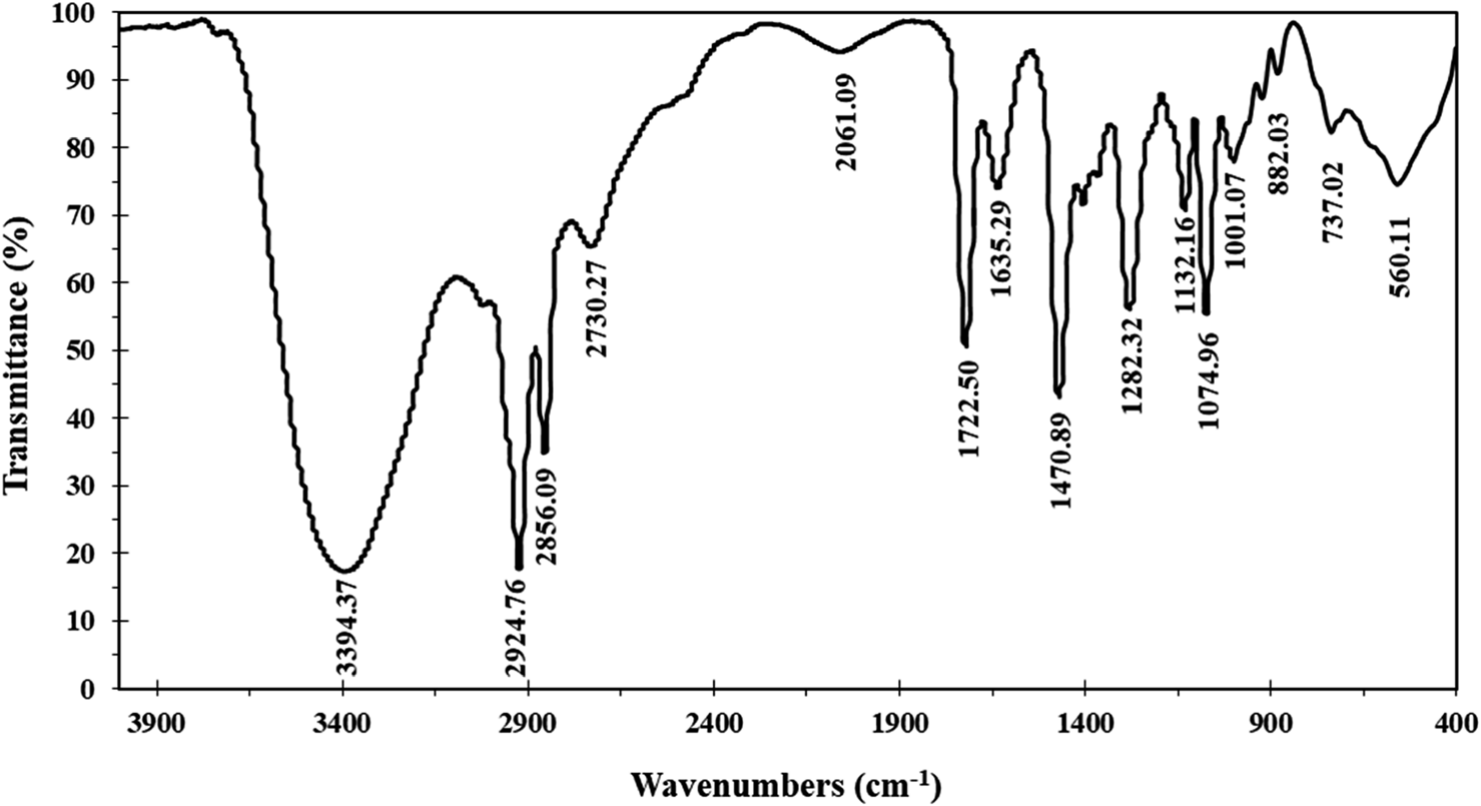
FTIR of the synthesized Schiff base.

The ionophore's FTIR spectrum (Fig. 1) was recorder within the range of 4000–400 cm^−1^ and it gave valuable information regarding the nature of the existing functional groups. The bands at 2924.76 cm^−1^ and 2856.09 cm^−1^ are assigned to (νCH) vibration, while the bands at 1282.32 cm^−1^, 1470.89 cm^−1^, and 737.02 cm^−1^ are assigned to (νCH_3_), (νCH_2_), and (ν(CH_2_)*n*–) vibrations, respectively. The observed signal at 1635.29 cm^−1^ is assigned to the (νC=N) stretching vibration which is a significant attribute of the Schiff base. The bands that appear at 1722.50 cm^−1^ and 1074.96 cm^−1^ represent (νC=O) and (νR_4_N) stretching vibrations, respectively. The broad band located at 3394 cm^−1^ is attributed to (νOH) group. The infrared spectroscopy confirmed the structure of the prepared ionophore.

**Fig. 2:**
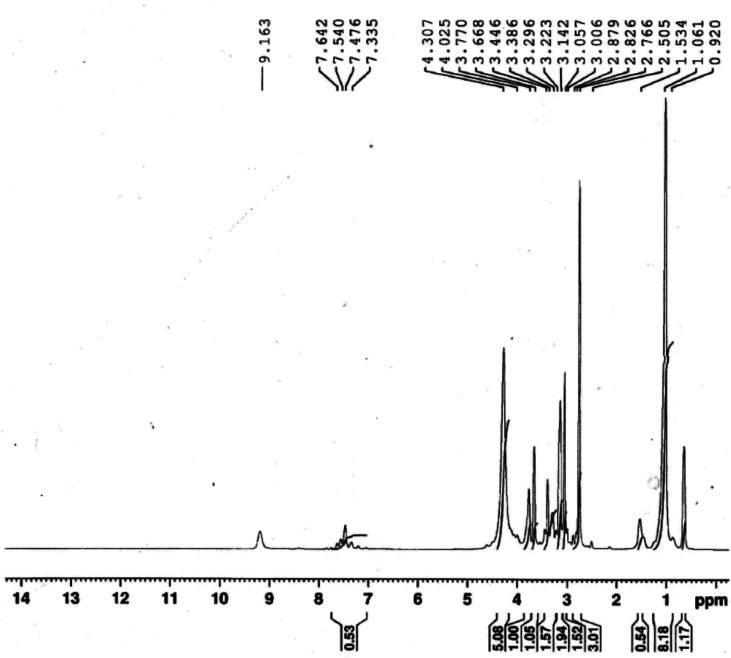
^1^HNMR of the synthesized Schiff base.

The ionophore's ^1^HNMR spectrum was recorded in DMSO solution as a solvent and is presented in Fig. 2. ^1^HNMR showed several characteristic chemical shifts. The triplet signal at *δ*  = 0.852 ppm is assigned to the terminal [–CH_3_] protons of the fatty chain (NCH_2_CH_2_(CH_2_)_9_C**H̲**_3_). The multiplet signal that is noticed at *δ* = 1.261 ppm is assigned to the protons of the repeated methylene groups in the fatty chain(NCH_2_CH_2_(C**H̲**_2_)_9_CH_3_). The signals of the methylene groups neighboring the nitrogen atom of the pyridine ring appeared as a multiplet at *δ* = 3.257 ppm for (NCH_2_C**H̲**_2_(CH_2_)_9_CH_3_), and as a triplet at *δ* = 3.868 ppm for (NC**H̲**_2_CH_2_(CH_2_)_9_CH_3_), respectively. The signal of the pyridine ring protons (Py-H) is observed at *δ* = 9.363 ppm as a singlet. The protons of the [–CH_3_] group adjacent to the azomethane group (N=C–C**H̲**_3_) came to resonance at *δ* = 3.026 ppm. The chemical shifts of the methyl groups attached to the pyrazol rings are observed at *δ* = 3.342 ppm and *δ* = 3.586 ppm for (NCC**H̲**_3_) and (NC**H̲**_3_), respectively. The aromatic ring protons (Ar–H) occurred at *δ* = 7.676 ppm as a doublet signal.

### 2.3. Sensor preparation and EMF measurements

PVC-based membranes of different compositions were prepared as previously described^18^*via* mixing thoroughly the appropriate amounts of the prepared ionophore, solvent mediators, and PVC. The typical weight ratio of the PVC to plasticizer was 1 : 2, and the percentages of Schiff base was changed from 0.5 to 4.0 wt%. Six different solvent mediators were used to manufacture the membranes: DOS, DBP, DOA, DOP, *o*-NPOE, and CN. Various quantities (0.25–1.00 wt%) of the anion excluder KTpClPB were added. The mixture was dissolved completely in THF (2 mL) then the membrane cocktail was transferred into a glass ring of 22 mm diameter above a smooth glass plate and left until THF was completely evaporated. The resulting transparent, flexible, homogenous with a uniform thickness membrane was used to produce smaller round shape working membranes of ∼5 mm diameter that was assembled on a sensor body filled with an internal solution of 1.00 × 10^−3^ mol L^−1^ of UO_2_(NO_3_)_6_. The same solution was used for overnight sensor conditioning prior to potential measurements to ensure equilibrium at the membrane-water interface.

The potential and pH observations were carried out at 25 ± 1 °C using an electrode-computer interface (Nico2000 Ltd., UK). The EMF readings were recorded for test solutions with uranium concentations from 1.00 × 10^−7^ to 1.00 × 10^−1^ mol L^−1^ using Ag/AgCl electrode as the reference electrode under constant pH and stirring rate. Inductively coupled plasma optical emission spectroscopy (ICP-OES, Thermo-iCAP6500, Japan) was utilized to determine uranium content in the water samples.

## 3. Results and discussion

**Scheme 2:**
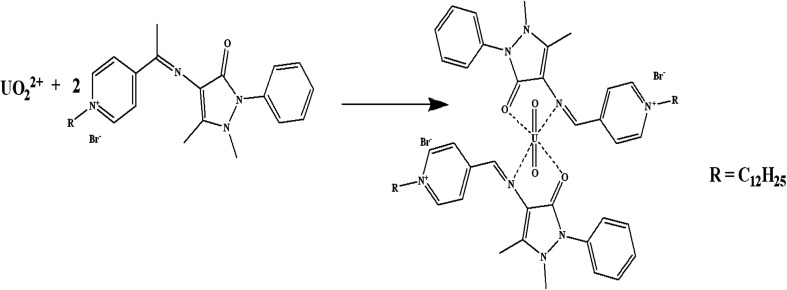
The proposed interaction mechanism of the synthesized Schiff base and uranyl ion.

Schiff bases have effective binding sites to recognize metal ions *via* the azomethine nitrogen. Schiff bases form stable complexes with uranium(vi) ions as they offer a well-suited binding pocket to accommodate the size and the preference for the coordination geometry of the U(vi) center in the UO_2_^2+^ ion.^29^ The preliminary experiments, showed that the fabricated sensor using the synthesized Schiff base has a sensitive response and good selectivity towards uranium(vi) ions. The possible interaction mechanism can be considered as that the Schiff base is bound to the uranyl cation centre in a bidentate fashion in the equatorial plane,^30^*i.e.* complexation between the N and O atoms in the synthesized Schiff base and U(vi) to form UO_2_L_2_ complex (Scheme 2). Similar results were obtained for uranium binding with other Schiff bases.^30,31^

### 3.1. Sensors' composition and performance attributes

The sensor's potential stability, sensitivity, and selectivity are significantly associated with the nature and composition of the membrane ingredients. Thus, the influence of various components, such as solvent mediators, ionophore, and lipophilic additives on the responses of the fabricated sensors was inspected as shown in Table 1.

**Table 1:** Optimization of the sensor composition

Membrane no.	Membrane composition (wt%)				Analytical parameters		
	Ionophore	PVC	Plasticizer	KTpClPB	Slope, mV per decade	Linear range, mol L^−1^	Response time, s
1	0.00	33.5	*o*-NPOE, 66.50	0.00	10.28	1.00 × 10^−3^ to 1.00 × 10^−1^	21
2	1.00	33.00	DOP, 66.00	0.00	21.30	1.00 × 10^−5^ to 1.00 × 10^−1^	12
3	1.00	33.00	DOA, 66.00	0.00	22.14	5.00 × 10^−6^ to 1.00 × 10^−2^	13
4	1.00	33.00	DOS, 66.00	0.00	20.05	5.26 × 10^−5^ to 1.00 × 10^−1^	17
5	1.00	33.00	DBP, 66.00	0.00	21.27	5.00 × 10^−6^ to 1.00 × 10^−1^	15
6	1.00	33.00	CN, 66.00	0.00	19.28	5.00 × 10^−4^ to 1.00 × 10^−2^	20
7	1.00	33.00	*o*-NPOE, 66.00	0.00	25.48	5.00 × 10^−6^ to 1.00 × 10^−1^	12
8	0.50	33.50	*o*-NPOE, 67.00	0.00	23.81	5.00 × 10^−6^ to 1.00 × 10^−1^	15
9	2.00	33.00	*o*-NPOE, 65.00	0.00	27.88	5.00 × 10^−6^ to 1.00 × 10^−1^	11
10	4.00	32.00	*o*-NPOE, 64.00	0.00	27.13	5.00 × 10^−6^ to 1.00 × 10^−1^	10
11	2.00	32.50	*o*-NPOE, 65.25	0.25	28.21	5.26 × 10^−6^ to 1.00 × 10^−1^	10
12	2.00	32.50	*o*-NPOE, 65.00	0.50	29.85	1.00 × 10^−6^ to 1.00 × 10^−1^	9
13	2.00	32.25	*o*-NPOE, 65.00	0.75	28.91	1.00 × 10^−6^ to 1.00 × 10^−1^	9
14	2.00	32.50	*o*-NPOE, 64.50	1.00	31.51	1.00 × 10^−6^ to 1.00 × 10^−1^	9

It is known that solvent mediators have a key role in controlling the sensor's working concentration range, detection limit, stability, and shelf life. Generally, the applied solvent mediator should possess elevated molecular mass and lipophilicity to adequately stabilize the membrane. Additionally, the solvent mediator should be capable of dissolving the ionophore as well as lipophilic salts present in the membrane.^32,33^ Among the six tested solvent mediators, namely *o*-NPOE, DOS, DOA, DOP, CN, and DBP, added in same ratio to the membrane; *o*-NPOE exhibited the best response. It is clear that solvent mediators' viscosity and dielectric constants affect the membrane's dielectric constant as well as the Schiff base's mobility and ion exchange in the membrane phase. Membranes having solvent mediators with low dielectric constants showed lower slopes due to the aggregation and reduced mobility of the ionophore^34^ which led to its reduced capability to form uranium complexes. However, the membrane includes a polar solvent mediator with a high dielectric constant, *o*-NPOE (*ε* = 24), exhibited the best response due to the ionophore's better mobility and extraction ability of uranium(vi) from solution.^32,35^

The amount of the ionophore in the membrane affects the sensor's response mechanism, therefore, different amounts of the prepared Schiff base were tested. The sensor without ionophore does not exhibit a Nernstian response while better performance characteristics were obtained upon adding ionophore up to 2 wt% where a near-Nernstian response of 27.88 mV per decade was obtained. Further increasing of ionophore content doesn't show sensible improvement in the sensor's performance. The observed enhancement of the response time by increasing the ionophore content could be due to the increased amounts of the active sites in the membrane that affects the sensor's phase boundary potential as a result of having elevated activities of Schiff base-uranium ions complex in the membrane phase.^36^

**Fig. 3:**
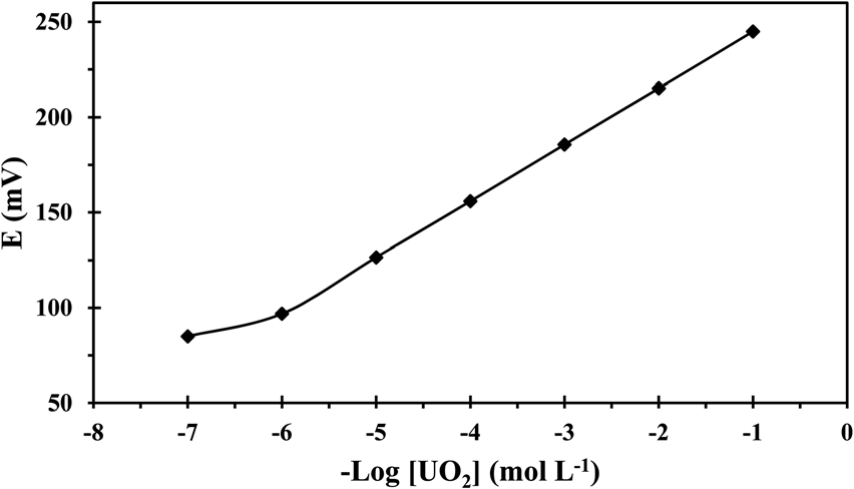
The calibration plot of the developed sensor.

Adding anion excluders to a cation-selective membrane enhances the sensor's selectivity and electrochemical performance as they diminish the anionic interference effects and enhance the sensor's extraction capability.^37^ Thus, the effect of lipophilic anions was considered and data given in Table 1 shows that the addition of KTpClPB enhanced the sensitivity values close to the theoretical response and reduced the response time through reducing the ohmic resistance. Considering this data and as seen in Table 1, the best performance relating to Nernestian slope, widest working concentration range, lowest detection threshold, and fastest response was exhibited by the composition 32.50% PVC, 65% NPOE, 2% ionophore, and 0.50% KTpClPB (membrane no. 12) and the calibration curve of this optomized membrane is depicted in Fig. 3.

**Fig. 4:**
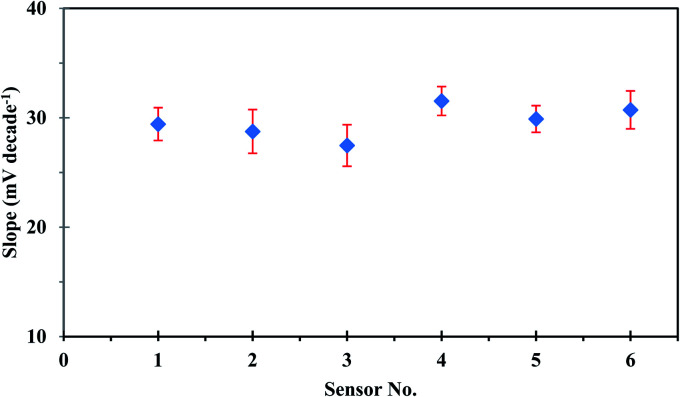
The reproducibility plot of the developed sensor.

The repeatability of the developed sensor was investigated using the same sensor in intraday calibration six times, and the sensor showed approximately similar linear ranges and sensitivities with an average slope of 29.53 ± 0.44 mV per decade. On the other hand, reproducibility studies were carried out to evaluate the performance of six independent sensors under the same working conditions. The sensor response was investigated by measuring the potential in uranium(vi) ion concentration in the range of 1.00 × 10^−1^ to 1.00 × 10^−6^ mol L^−1^. The sensors showed good reproducibility with a relative standard deviation of 4.86%. The observed difference in the slope values of different sensors, as could be seen in Fig. 4, originates from the fluctuation in uranium extraction equilibrium as a result of the variation of the membrane thickness at different locations.^38^

It is worth mentioning that the sensor's performance was explored in relation to concentrations of the filling solution (1.00 × 10^−2^ to 1.00 × 10^−4^ mol L^−1^) and equilibration time (1–48 h). The results showed that the filling solution influences the cell's potential stability to some degree where 1.00 × 10^−3^ mol L^−1^ was suitable for the sensor's smooth functioning while 24 h was appropriate to generate stable and reducible potentials with uranium(vi) solutions.

### 3.2. Optimization of the working pH

**Fig. 5:**
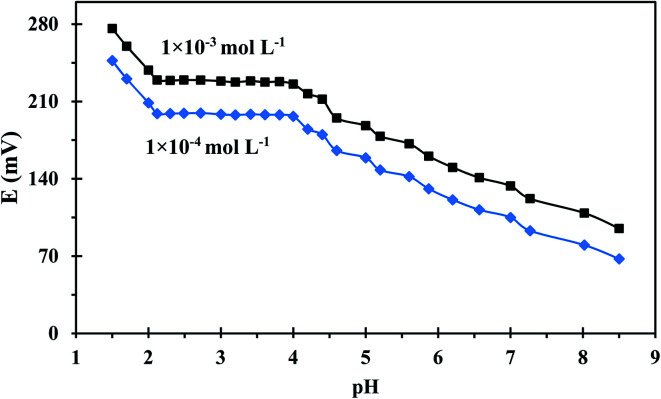
Potential-*versus*-pH trace for the developed sensor.

pH is a significant factor to be considered during uranium measurements because the change in the solution's pH can strongly affect uranium speciation, solubility, and complexation with the ionophore. The pH dependence of the developed sensor was evaluated in a pH range of 1.50–8.50 using two different uranium(vi) concentrations and the results were illustrated in Fig. 5. The sensor's potential response is not influenced by pH changes over the range 2.10–4.21, thus this range is recommended for potential measurement. At lower pHs, the sensor response changed dramatically due to the presence of excess H^+^ ions which resulted in the protonation of the N atoms of the employed ionophore. On other hand, the diminished response at higher pHs is ascribable to uranium hydrolysis and the formation of low-affinity uranium species in the solution.^39^ A pH of 3 was used for further potential measurements as was recommended in the literature.^40^

### 3.3. Dynamic response time

**Fig. 6:**
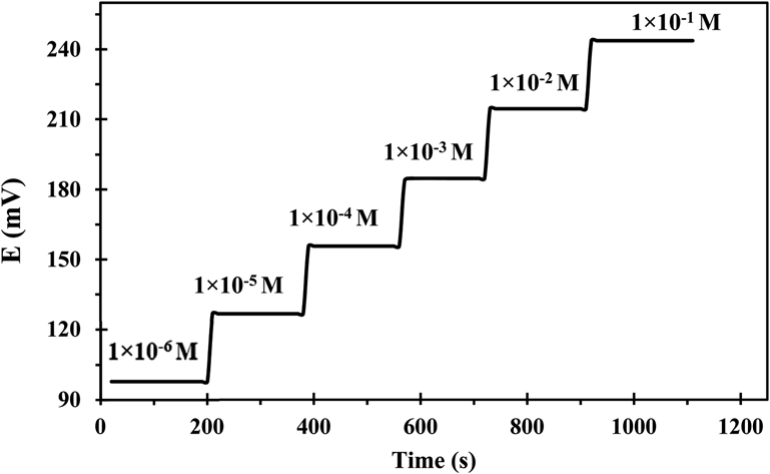
Potential-*versus*-time trace for the developed sensor at various uranium(vi) concentrations.

Response time is the operating parameter that informs how long it takes for the sensor to reach stable potential readings. The dynamic response time was determined when the potential had become constant after immersing the developed sensor in various successive concentrations of uranium(vi) solutions each having a tenfold difference in concentration (1.00 × 10^−1^ to 1.00 × 10^−6^ mol L^−1^). The obtained results were depicted in Fig. 6 which revealed that the developed sensor has a fast response even to low uranium(vi) concentrations. The mean time needed by the suggested sensor for attaining a steady potential response was 9 s over the whole concentration range. The notable fast dynamic response time is attributed to the rapid complexation–decomplexation exchange kinetics between uranium(vi) ions and the ionophore at the membrane-solution boundary interface.^41^

The useful sensor lifetime was investigated over a period of 16 weeks; by periodically conducting calibration and calculating the calibration curve slope and detection limit values. The obtained results as depicted in Table 2 revealed no significant difference in the slope value during the first 8 weeks. Afterward, a dramatic deterioration of the slope and detection limit was noted. This could be possibly attributed to the leaching of membrane ingredients into the sample solution during use.^42^ Additionally, the membrane swelling led to a fragile sensor close to its lifespan termination.

**Table 2:** Lifetime of the developed sensor

Week	Slope, mV per decade	Detection limit, mol L^−1^
1	29.59	3.90 × 10^−7^
2	29.92	3.72 × 10^−7^
3	28.97	3.15 × 10^−7^
4	30.11	3.58 × 10^−7^
5	29.22	4.30 × 10^−7^
6	28.83	1.08 × 10^−6^
8	27.18	2.51 × 10^−6^
10	25.13	5.25 × 10^−6^
12	20.78	1.13 × 10^−5^
14	18.92	3.28 × 10^−5^
16	15.78	1.56 × 10^−4^

### 3.4. Interference studies

For real samples application, the sensor's selectivity is a vital characteristic as the samples might contain various interfering species that hinder the analyte ions and result in errors during the analysis. The selectivity behavior of the developed sensor was investigated employing the separate solution method (SSM).^43^ According to this method the potential readings (mV) were measured for a series of interfering ions and uranium(vi) ions in a concentration range of 1.00 × 10^−6^ to 1.00 × 10^−1^ mol L^−1^. The selectivity coefficients log (log *K*^pot^_U(VI),*x*_), which describe the sensor's ability to discriminate between uranium(vi) ions and other ions in the solution, were calculated from the EMF values obtained at 1.00 × 10^−1^ mol L^−1^ assuming theoretical slopes.^16^ The calculated potentiometric selectivity coefficient values for the prepared sensor are represented in Table 3. The negative values of the selectivity coefficients imply the high selectivity of the developed sensor towards uranium(vi) ions. This indicates the absence of significant interference from the investigated cations in the sensor performance, thus the sensor can adequately act as selective to uranium(vi) ions.

**Table 3:** Selectivity coefficients of various interfering ions for the developed sensor

Interfering ion (*x*)	log *K*^SSM^_U(VI),*x*_	Interfering ion (*x*)	log *K*^SSM^_U(VI),*x*_
La^3+^	−3.85	Na^+^	−4.31
Al^3+^	−3.59	Li^+^	−4.12
Cr^3+^	−3.36	NH_4_^+^	−3.75
Fe^3+^	−2.06	K^+^	−3.45
Co^2+^	−4.25	Cs^+^	−2.19
Ba^2+^	−4.12	Bi^2+^	−4.26
Sr^2+^	−4.18	Cd^2+^	−4.41
Ca^2+^	−3.52	Mn^2+^	−4.07
Zn^2+^	−3.63	Mg^2+^	−3.39
Pb^2+^	−3.89	Cu^2+^	−3.80
Hg^2+^	−4.75	Ni^2+^	−4.27

### 3.5. Analytical applications

The sensor's utility was investigated thorough the direct uranium assay in water samples that were used without previous treatment. A known amount of uranium(vi) was spiked to each individual sample and determined by the developed sensor after constructing a calibration curve. ICP-OES measurements were also performed, as a reference technique, to assess the results' accuracy. The data acquired from three replicate analyses using the same sensor are summarized in Table 4. The contents of measured uranium(vi) ions agreed satisfactorily with those determined by ICP-OES and showed good recoveries for all samples. This indicates the utility of the developed sensor to measure uranium(vi) content in real samples.

**Table 4:** Analysis of uranium(vi) content in spiked samples

Sample no.	Concentration^a^ (ppm)			
	Added	Found by ICP-OES	Found by the developed sensor	Recovery ± RSD (%)
1	30	30.15 ± 0.34	30.58 ± 0.43	101.95 ± 1.78
2	50	50.51 ± 0.35	51.31 ± 0.51	102.62 ± 1.26
3	75	75.66 ± 0.33	74.62 ± 1.11	99.49 ± 1.82
4	100	99.89 ± 0.26	98.69 ± 0.98	98.70 ± 1.20

a Average of three measurements ± standard deviation.

### 3.6. Comparison of the developed sensor with uranium(vi) sensors previously reported in literature

A comparison of the performance characteristics of the developed sensor with previously reported uranium potentiometric sensors was made in the terms of sensitivity, dynamic range, detection limit, response time, and useable pH range. The developed sensor based on the synthesized Schiff base revealed potentiometric characteristics which are comparable to or superior in many respects than those reported in literature (Table 5).

**Table 5:** Comparison of some response features of the developed sensor and reported uranium(vi) sensors based on organic ionophores

Ionophore	Slope	Response time (s)	pH	Linear range (mol L^−1^)	Detection limit (mol L^−1^)	Reference
5,11,17,23-Tetra-*tert*-butyl-25,27-bis(hydroxy)-26-(ethoxycarbonylmethoxy)-28-(diethyl carbamoyl-methoxy) calix[4]arene	28.6	<30	5.5–8.5	5.0 × 10^−6^ to 1.0 × 10^−1^	3.0 × 10^−6^	13
5,11,17,23,29,35-Hexa-*tert*-butyl-37,38,39,40,41,42-hexahydroxy calix[6]arene and tri-*n*-octyl phosphine oxide	27.0	30	3.2–4.6	1.0 × 10^−1^ to 10	NA	14
Benzo-15-crown-5	29.5	∼15	4.0–7.0	1.0 × 10^−4^ to 1.0 × 10^−1^	1.0 × 10^−4^	15
Dibutyl butyl phosphonate	28.6	∼30	2.1–3.4	5.0 × 10^−6^ to 1.0 × 10^−1^	3.0 × 10^−6^	16
Di-*n*-octyl phenyl phosphonate	29.7	∼30	2.1–3.4	5.5 × 10^−5^ to 1.0 × 10^−1^	1.2 × 10^−5^	16
Dimethylsuphoxide	30.0	15	1.5–4.0	1.0 × 10^−7^ to 1.0 × 10^−1^	8.9 × 10^−8^	17
1,18-Diaza-3,4;15,16-dibenzo-5,8,11,14,21,24-hexaoxacyclohexaeicosane-2,17-dione	29.8	<12	3.0–3.5	3.0 × 10^−6^ to 8.2 × 10^−3^	2.2 × 10^−6^	19
2,2′-[1,2-Ethandiyl bis(nitriloethylidene)]bis(1-naphthalene)	28.5	<20	3.0–4.0	1.0 × 10^−7^ to 1.0 × 10^−1^	7.0 × 10^−8^	21
*N*,*N*′-4,5-(Propylenedioxy)benzenebis(3,5-di-*tert*-butylsalicylideneimine)	28.8	∼20	1.0–5.0	1.0 × 10^−6^ to 1.0 × 10^−2^	6.5 × 10^−7^	22
*N*,*N*′-4,5-(Ethylenedioxy)benzenebis(salicylideneimine)	28.0	<60	1.5–4.0	1.0 × 10^−6^ to 1.0 × 10^−2^	3.2 × 10^−7^	23
Bis(2-hydroxyacetophenone)ethylenediimine	29.3	<5	3.0–4.5	5.0 × 10^−6^ to 5.0 × 10^−2^	2.0 × 10^−6^	24
4-(1-((1,5-Dimethyl-3-*oxo*-2-phenyl-2,3-dihydro-1*H*-pyrazol-4-yl)imino)ethyl)-1- dodecylpyridin-1-ium bromide	29.8	9	2.1–4.2	1.0 × 10^−6^ to 1.0 × 10^−1^	3.9 × 10^−7^	This work

## 4. Conclusions

In this work, the potentiometric assay of uranium(vi) ions in water samples was successfully achieved using newly prepared Schiff base derivative as an ionophore. The developed sensor is characterized by good analytical parameters including selective response towards uranium(vi) ions, good sensitivity, rapidity, reproducibility, wide linearity, and reasonable pH range. The sensor responded to uranium(vi) ions in a Nernstian fashion and was successfully applied to the direct uranium determination in various spiked water samples. The developed sensor represents a convenient option for uranium(vi) measurements in real samples without requiring preconcentration or pre-treatment steps.

## Conflicts of interest

There are no conflicts to declare.

## Supplementary Material
